# Physiological and Subjective Measures Associated with Withdrawal from Intravenous Sedation in Dental Phobia: A Prospective Cohort Study

**DOI:** 10.3390/jcm15020614

**Published:** 2026-01-12

**Authors:** Yukihiko Takemura, Yoshiharu Mukai, Toshiya Morozumi, Kyoko Arai, Ryo Wakita, Ayako Mizutani, Atsushi Matsumoto, Takuro Sanuki

**Affiliations:** 1 Department of Restorative Dentistry, Kanagawa Dental University, Yokosuka 238-8580, Japan; mukai@kdu.ac.jp; 2 Department of Endodontics, The Nippon Dental University School of Life Dentistry at Niigata, Niigata 951-8580, Japan; morozumi@ngt.ndu.ac.jp (T.M.); araik@ngt.ndu.ac.jp (K.A.); 3 Department of Dental Anesthesiology, Kanagawa Dental University, Yokosuka 238-8580, Japan; wakita@kdu.ac.jp (R.W.); ayako07mizutani16@jcom.zaq.ne.jp (A.M.); 4 Sugita Orthodontics and Pediatric Dentistry, Yokohama 235-0036, Japan; 39matsushi@gmail.com; 5 Department of Clinical Physiology, Institute of Biomedical Sciences, Nagasaki University, Nagasaki 852-8523, Japan; sanuki-t@nagasaki-u.ac.jp

**Keywords:** dental phobia, intravenous sedation, salivary alpha-amylase, visual analog scale, sedation withdrawal, sympathetic–adrenal–medullary axis

## Abstract

**Background:** Patients with dental phobia frequently require intravenous sedation (IVS) to undergo dental treatment; however, some can gradually discontinue IVS through repeated clinical experiences. The physiological and psychological factors influencing successful IVS withdrawal remain unclear. This study aimed to compare physiological (sAA, HR) and subjective (VAS) measures between patients who discontinued IVS and those who remained dependent on IVS. **Methods:** This prospective cohort study included 51 patients with dental phobia treated under IVS. Participants were classified into a Non-Sedation Group (NSG; *n* = 25) and a Sedation-Dependent Group (SDG; *n* = 26) based on their ability to discontinue IVS during the course of treatment. Salivary alpha-amylase (sAA), heart rate (HR), and visual analog scale (VAS) scores for fear, tension, and anxiety were assessed at predefined time points from the waiting room to venous cannulation. Treatment satisfaction and expectations for future treatment were also evaluated. **Results:** sAA activity was significantly higher in the SDG than in the NSG at T0 and T1 (*p* < 0.05), indicating higher levels of selected physiological measures during anticipatory phases; however, the difference at T2 was not significant. HR differed significantly only in the waiting room, whereas no between-group differences were observed in self-reported VAS scores for fear, tension, or anxiety at any time point, indicating a dissociation between physiological and subjective stress measures. Treatment satisfaction and expectations for future treatment were significantly higher in the SDG. **Conclusions:** Patients who remained dependent on IVS showed higher levels in selected physiological measures at the group level during anticipatory stages, whereas no corresponding differences were observed in self-reported subjective measures. These findings are exploratory and descriptive in nature and do not imply predictive or causal relationships.

## 1. Introduction

Dental phobia is a severe and persistent condition characterized by marked fear or distress related to dental procedures or associated stimuli, often accompanied by avoidance behavior and functional impairment [1]. Dental phobia is clinically distinguished by its severity and impact on daily life. Epidemiological studies have reported that approximately 10–15% of adults may experience severe dental anxiety, including phobic fear, depending on the population and assessment methods used [2], suggesting that it represents a relevant public health concern. Avoidance of dental treatment among individuals with dental phobia is associated with oral health deterioration and a subsequent decline in quality of life [3,4].

Various factors can elicit fear and anxiety in patients. Sensory stimuli, such as the sounds of drilling and suction [1], smell of dental materials or chemicals [5], visual exposure to needles or sharp instruments [6], and sensations of vibration or pressure during treatment [7], may trigger anxiety. Moreover, psychological factors, including fear of pain [8], perceived loss of control while seated in the dental chair [9], and tension associated with pre-treatment waiting [10], can exacerbate these reactions. Collectively, these sensory and psychological factors contribute to heterogeneous physiological and subjective stress-related responses, whose relative expression may vary across individuals depending on prior experiences, cognitive appraisal, and coping strategies.

When an individual is exposed to a fearful situation, emotional neural circuits involving the amygdala are thought to be engaged, which may lead to activation of autonomic and endocrine systems via the hypothalamus [11]. The sympathetic–adrenal–medullary (SAM) axis rapidly releases adrenaline and noradrenaline, leading to physiological responses such as increased heart rate (HR), elevated blood pressure, and sweating [12]. In contrast, the hypothalamic–pituitary–adrenal (HPA) axis responds more slowly and secretes cortisol to sustain a prolonged stress response [13]. Salivary alpha-amylase (sAA) has been widely used as a non-invasive marker of SAM axis activation to assess acute stress-related responses in various clinical and experimental settings [14,15]. Although sAA has been reported to correlate with stress-related responses, its clinical significance and relationship with sedation-related outcomes remain unclear.

Intravenous sedation (IVS) is an important technique in dental practice that allows safe treatment of patients experiencing intense dental fear or panic reactions while avoiding the use of general anesthesia [16]. Benzodiazepines exert sedative and anxiolytic effects through the γ-aminobutyric acid type A (GABA_A) receptor [17], lowering the level of consciousness and inducing anterograde amnesia. While IVS has been shown to reduce acute distress and facilitate treatment completion in many patients, it primarily functions as a symptomatic intervention, and fear responses may persist or re-emerge after the pharmacological effects subside [18]. Moreover, owing to potential adverse effects such as respiratory depression, IVS is not appropriate for all patients [19]. In addition, pharmacologic sedation may restrict opportunities for exposure to fear-eliciting stimuli, potentially impeding long-term fear reduction [20]. Consequently, the development of multifaceted strategies that integrate pharmacological and psychological interventions has been emphasized.

The concept of desensitization, which involves gradually familiarizing patients with fear-provoking treatment situations, represents one such approach. Systematic desensitization, which combines graded exposure to feared stimuli with relaxation techniques, has been adapted to dentistry as a psychological therapy for reducing fear responses [21]. However, for patients requiring IVS, the initial intensity of their phobia often precludes direct exposure to fear-eliciting stimuli, making the early stages of desensitization therapy challenging to implement. Therefore, identifying the factors that enable a safe transition from IVS to non-sedated treatment is a critical first step toward broader psychological interventions [22]. Clinically, some patients can continue treatment without IVS after initial sedation, whereas others remain dependent on IVS for subsequent treatments. Despite the clinical relevance of IVS withdrawal, prior studies have largely examined dental fear, anxiety, or stress-related biomarkers in isolation, without simultaneously considering physiological measures, subjective emotional experiences, and behavioral–clinical outcomes within the context of real-world IVS treatment courses.

Despite the widespread clinical use of IVS in patients with dental phobia, it remains unclear whether physiological stress-related measures, subjective emotional experiences, and clinically observed treatment trajectories show concordant or dissociated patterns across different phases of real-world IVS treatment courses. Importantly, existing studies have not clarified how such potential dissociations manifest along actual treatment courses, nor have they established whether these measures align with clinically observed outcomes such as continuation or discontinuation of IVS. The present study does not aim to resolve causal mechanisms, predictive relationships, or determinants of IVS withdrawal; rather, it addresses this gap at a minimal descriptive level by systematically documenting phase-specific patterns of physiological measures (sAA and HR), subjective VAS ratings, and observed clinical treatment courses in a single IVS-treated dental phobia cohort, without presupposing concordance across outcome domains. By doing so, the study provides a structured, phase-specific description of where physiological measures and subjective ratings converge or diverge along the IVS treatment course, which has been insufficiently documented in prior IVS-focused dental phobia cohorts.

In this study, all psychological assessments were conducted in Japanese and targeted Japanese patients, and the terminology was therefore used in accordance with common Japanese clinical distinctions. “Dental phobia” refers to the clinical condition defining the study population. In contrast, “fear” denotes an emotional response directed toward specific and identifiable dental stimuli, whereas “anxiety” refers to a more diffuse anticipatory emotional state without a clearly defined source. The term “tension” was used to describe a subjective psychological and bodily state of readiness or tightness that commonly accompanies anticipation of dental treatment. These terms were treated as state-dependent subjective experiences rather than diagnostic categories.

This study aimed to compare patients with dental phobia who underwent dental treatment under IVS and were subsequently able to continue treatment without IVS with those who remained dependent on IVS. The objective was to descriptively compare the two groups using sAA activity, HR, and visual analog scale (VAS) scores as physiological and subjective measures, without assuming explanatory or predictive relationships.

## 2. Materials and Methods

### 2.1. Study Design and Participants

This prospective cohort study was conducted at Kanagawa Dental University Hospital. A total of 121 patients were clinically referred for dental treatment under IVS between January 2018 and December 2024. Of these, 46 patients were excluded prior to enrollment based on predefined criteria, including age younger than 18 or older than 80 years, American Society of Anesthesiologists physical status ≥III, cognitive impairment preventing questionnaire completion, pronounced gag reflex as the primary complaint, a history of white-coat hypertension, or CMDAS scores below 20.

**Figure 1:**
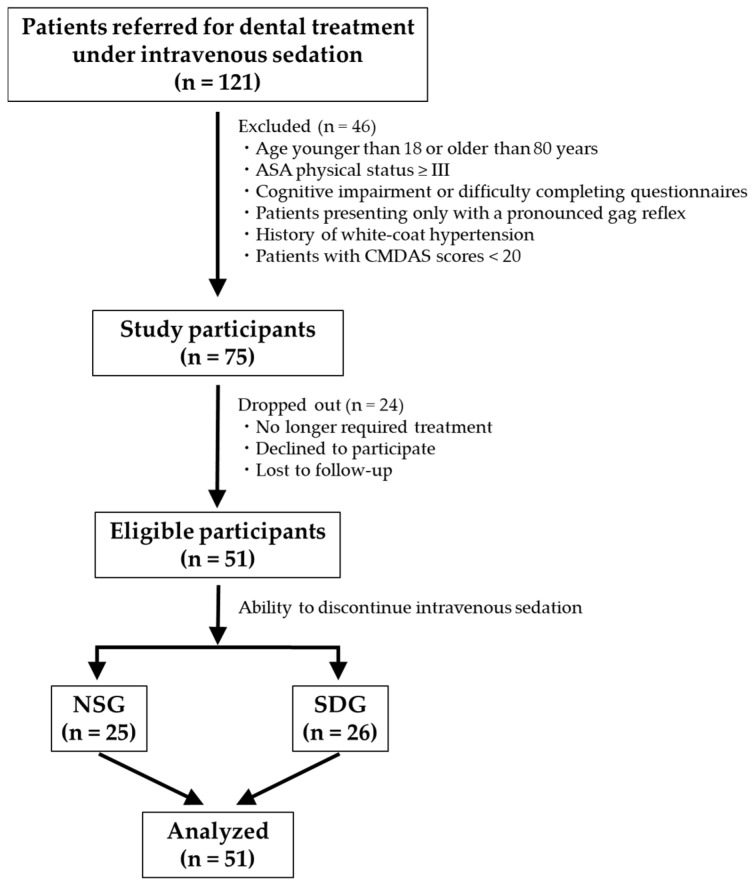
Study flow chart illustrating patient recruitment and allocation to study groups. After screening, eligible patients were allocated to the non-sedation group (NSG) or the sedation-dependent group (SDG) according to their clinical treatment course, specifically based on the presence or absence of withdrawal from intravenous sedation.

Among the remaining 75 eligible patients, 24 were subsequently excluded because they no longer required dental treatment, declined participation, or were lost to follow-up. Consequently, 51 patients were included in the final analysis and were classified into a non-sedation group (NSG) or a sedation-dependent group (SDG) based on their clinical treatment course and ability to discontinue IVS (Figure 1).

Group classification was based on observed treatment behavior and clinical outcomes rather than patient self-report alone. Patients were classified into the NSG if they were able to complete supportive periodontal therapy (SPT) sessions without pharmacological sedation, accept the use of dental instruments, and complete treatment without interruption or overt anxiety-related behaviors. In contrast, patients were classified into the SDG if they required IVS for all dental treatment sessions and were unable to complete any session without sedation.

All participants provided written informed consent prior to study participation. Group classification was applied post hoc for descriptive and exploratory purposes and was not based on predefined predictive criteria established before treatment initiation. Accordingly, the NSG and SDG should be interpreted as outcome-based categories reflecting the clinical treatment course, rather than as etiological, prognostic, or risk stratification groups (Table 1). The number, frequency, and duration of IVS sessions differed across patients according to individual clinical needs and treatment course. For this reason, IVS exposure was not predefined or standardized as a study variable.

The Customized Modified Dental Anxiety Scale (CMDAS), which was developed based on the Modified Dental Anxiety Scale (MDAS), was used to assess the severity of dental anxiety for screening purposes [23]. The CMDAS consists of five items, each rated on a five-point scale ranging from 1 (not anxious at all) to 5 (extremely anxious), yielding a total score of 25 points. In a preliminary observational assessment conducted prior to this study, patients with CMDAS scores of 20 or higher consistently exhibited marked anxiety-related behaviors at the time of dental treatment. In all such patients, the use of IVS was clinically judged to be necessary to safely perform dental procedures. In contrast, among patients who exhibited similar anxiety-related behaviors but had CMDAS scores of 19 or lower, some cases could be managed without IVS. Based on these clinical observations, a CMDAS score of 20 or higher was adopted in the present study not as a diagnostic criterion, but as an operational inclusion criterion to define a patient group clinically characterized by severe dental fear requiring IVS (Table 2). [24]

The exclusion criteria were as follows: (1) age younger than 18 or older than 80 years; (2) American Society of Anesthesiologists (ASA) physical status classification ≥III; (3) cognitive impairment or inability to complete questionnaires; (4) patients presenting only with a pronounced gag reflex; and (5) a history of white-coat hypertension, defined as transient blood pressure elevation in clinical settings without a diagnosis of chronic hypertension, which may influence autonomic stress responses. Patients with CMDAS scores < 20 or those scoring ≥ 20 but not exhibiting observable anxiety-related behaviors were also excluded.

### 2.2. Environmental Conditions

IVS was administered by experienced anesthesiologists using commonly employed sedative agents, with drug selection, dosing, and titration individualized according to each patient’s clinical condition, treatment requirements, and intraoperative responses. Because of this individualized approach, sedation protocols were not standardized or predefined as study variables within this clinical cohort.

In this study, to account for potential habituation effects associated with repeated treatments, the analyses focused on the final dental treatment session performed under IVS after multiple IVS-assisted procedures. Psychological and physiological assessments were conducted by the same operator following standardized explanations.

**Figure 2:**
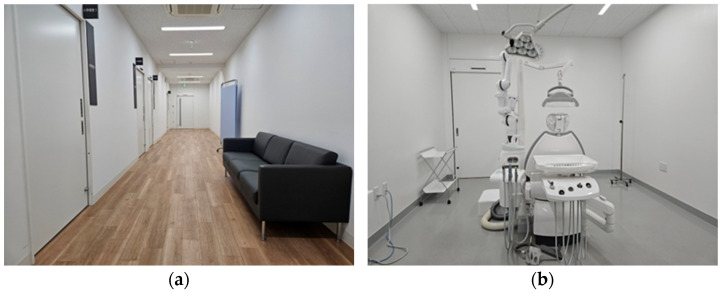
Standardized clinical environment: (**a**) waiting room and (**b**) treatment room. Walls were uniformly white, and natural light was eliminated to control environmental factors.

All evaluations were performed in the same waiting room and dental operatory under controlled conditions. The illumination, ambient noise, and room temperature were kept constant throughout the assessments. Specifically, both the waiting and treatment rooms were illuminated with neutral-white fluorescent lighting, maintained at a room temperature of 25–27 °C, and insulated from background noise. The walls were painted white, and no windows were present, eliminating the influence of natural light and ensuring uniform evaluation conditions for all the sessions (Figure 2). The standardization of these environmental conditions was intended to minimize variability in situational stressors during measurement.

### 2.3. Evaluation Parameters and Measurement Timings

**Figure 3:**
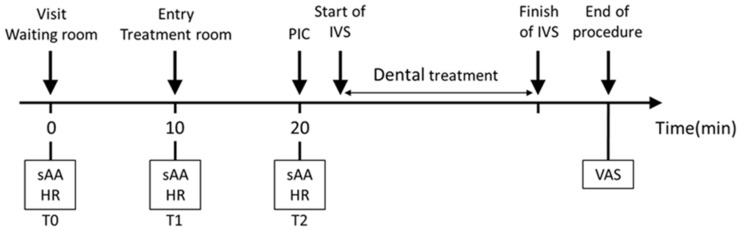
Study protocol and time points for measurement. sAA: salivary alpha-amylase; HR: heart rate; PIC: peripheral intravenous cannulation; VAS: visual analog scale; and IVS: intravenous sedation. T0: Upon being seated in the waiting room; T1: Immediately after entering the treatment room; and T2: Just before PIC.

The physiological parameters included sAA and HR, and the psychological parameters were assessed using VAS scores. sAA activity and HR were measured at three time points: (1) immediately after the patient was seated in the waiting room (T0, baseline), (2) immediately after entering the dental operatory (T1, evaluation of the response to environmental change), and (3) during peripheral venous cannulation (T2, when anticipatory anxiety was expected to be maximal). VAS scores were evaluated after treatment to assess fear, tension, and anxiety, as well as the intention to postpone future appointments, treatment satisfaction, and expectations for subsequent dental treatments (Figure 3).

To minimize circadian effects, all measurements were performed during daytime clinical hours under routine outpatient conditions. Information on current medication use and medical history was obtained from clinical records; however, no exclusions were made based solely on these factors. Given the non-interventional cohort design and clinical setting, complete control of all potential confounders was not feasible, and these factors should be considered when interpreting the results.

### 2.4. Measurement Methods

#### 2.4.1. Measurement of sAA Activity

sAA activity was measured using a dry clinical chemistry analyzer Salivary Amylase Monitor (NIPRO Corporation, Osaka, Japan) [25]. Unstimulated saliva was used for the measurement, and participants were instructed to refrain from eating, drinking, smoking, and exercising for at least 3 h before sample collection. The procedure is performed under resting conditions following standardized protocols. sAA was regarded as an immediate physiological stress marker that sensitively reflects sympathetic nervous system activity.

#### 2.4.2. Measurement of HR

HR was measured at rest using an automated clinical blood pressure and pulse monitor (Dental Moneo BP-A308D, Fukuda Denshi Co., Ltd., Tokyo, Japan). To minimize the influence of conversation and physical movement, the participants were kept at rest immediately before the measurement.

#### 2.4.3. Measurement of VAS

Psychological assessments were performed using VAS to evaluate patients’ subjective experiences. The VAS items for fear, anxiety, and tension were designed to capture state-dependent, self-perceived emotional and bodily experiences as verbally understood by Japanese patients, rather than diagnostic severity or trait-level characteristics.

(1) Fear (in the waiting room, upon entering the dental operatory, during intravenous cannulation, and recalling the next treatment).
(2) Tension (same four time points as above).
(3) Anxiety (same four time points as above).
(4) Intention to postpone future appointments.
(5) Treatment satisfaction.
(6) Expectations for subsequent dental treatment.

All psychological assessments were conducted by the same operator who provided standardized explanations to ensure consistency in the evaluations.

### 2.5. Statistical Analysis

The sample size was calculated using G*Power software (version 3.1.9.2; Heinrich Heine University Düsseldorf, Düsseldorf, Germany). Assuming a moderate effect size, a two-sided significance level of 0.05, and statistical power of 0.80, the required sample size for the primary outcomes was estimated to be 25 participants per group.

Comparisons of each parameter between the NSG and SDG groups were performed using the Mann–Whitney U-test. Given the non-interventional cohort design and the small sample size (NSG: *n* = 25, SDG: *n* = 26), which pose a risk of insufficient statistical power, the results should be interpreted as preliminary and hypothesis-generating. A *p*-value of <0.05 was considered statistically significant, and the Bonferroni correction was applied for multiple comparisons. Statistical analyses were conducted using EZR (Version 1.54, Saitama Medical Center, Jichi Medical University, 2023), a graphical user interface for R (The R Foundation for Statistical Computing, Vienna, Austria).

### 2.6. Ethical Considerations

This study was reported as a prospective cohort study in accordance with the STROBE guidelines [26]. This study was conducted in accordance with the Declaration of Helsinki and approved by the Ethics Committee of Kanagawa Dental University (Approval Nos. 554, 601, 615, and 937). This study was registered with the University Hospital Medical Information Network Clinical Trials Registry (UMIN-CTR; UMIN000038122). Written informed consent was obtained from all participants included in the study.

## 3. Results

Given the outcome-based group classification and the descriptive analytical framework of this cohort, and the presence of multiple outcome measures, the results should be interpreted with caution.

### 3.1. Measurement of sAA Activity

**Figure 4:**
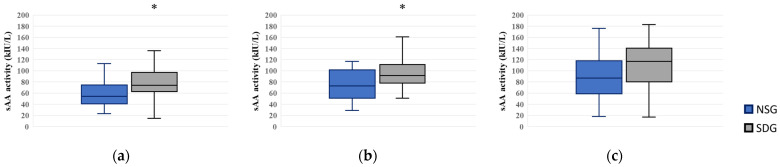
Box plots of salivary alpha-amylase (sAA) activity at three time points. NSG: Non-Sedation Group; SDG: Sedation-Dependent Group. (**a**) T0 (baseline in the waiting room), (**b**) T1 (immediately after entering the treatment room), and (**c**) T2 (just before peripheral intravenous cannulation). Boxes represent the interquartile range (IQR); the horizontal line within each box indicates the median, and the whiskers show the minimum and maximum values. An asterisk (*) denotes a statistically significant difference between the groups (*p* < 0.05).

At T0, salivary α-amylase activity was 54.0 (40.5–74.5) in the NSG and 74.0 (62.5–97.0) in the SDG, with the SDG exhibiting significantly higher values (*p* = 0.007) and a moderate between-group effect size (*r* = 0.38, 95% CI: 0.10–0.61), indicating a clinically meaningful difference. At T1, salivary α-amylase activity was 73.0 (51.0–101.5) in the NSG and 91.5 (78.0–111.3) in the SDG; the between-group difference was statistically significant (*p* = 0.047) and showed a moderate effect size (*r* = 0.30, 95% CI: 0.03–0.52). At T2, sAA activity was 87.0 (59.0–118.0) in the NSG and 117.0 (80.3–140.5) in the SDG; although the median was higher in the SDG, this difference was not statistically significant (Figure 4).

Absolute sAA values varied considerably among individuals; therefore, the analysis focused on between-group differences rather than comparisons with normative thresholds.

### 3.2. Measurement of HR

**Figure 5:**
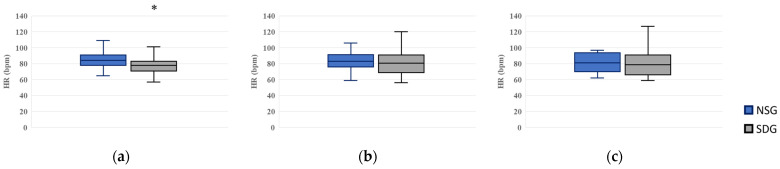
Box plots of heart rate (HR) at three time points. NSG: Non-Sedation Group; SDG: Sedation-Dependent Group. (**a**) T0 (baseline in the waiting room), (**b**) T1 (immediately after entering the treatment room), and (**c**) T2 (just before peripheral intravenous cannulation). Boxes represent the interquartile range (IQR); the horizontal line within each box indicates the median, and the whiskers show the minimum and maximum values. An asterisk (*) denotes a statistically significant difference between the groups (*p* < 0.05).

At T0 (waiting room), HR was higher in the NSG than in the SDG (*p* = 0.024), with a moderate between-group effect size (*r* = 0.34, 95% CI: 0.08–0.59). At T1, HR was 83.0 (76.0–91.5) in the NSG and 80.5 (68.8–91.0) in the SDG; at T2, HR was 81.0 (70.0–93.5) in the NSG and 78.5 (66.0–91.0) in the SDG. No statistically significant differences in HR were observed between the groups at T1 or T2 (Figure 5).

### 3.3. Measurement of VAS

#### 3.3.1. Measurement of Fear Scores

**Figure 6:**
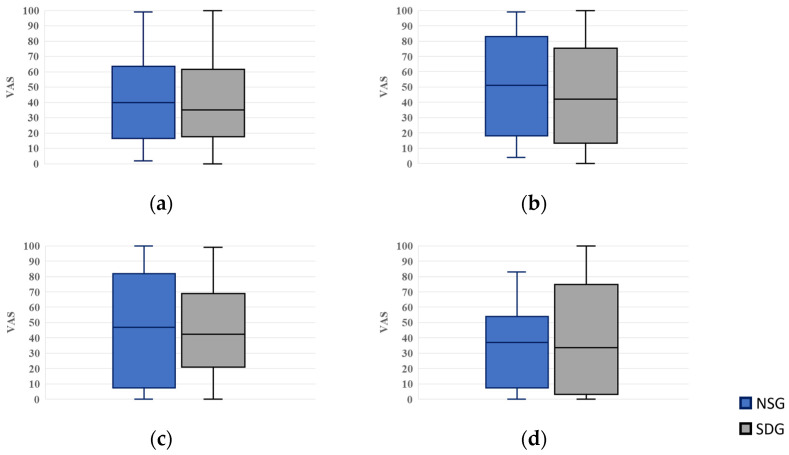
Box plots of visual analog scale (VAS) scores for fear. NSG: Non-Sedation Group; SDG: Sedation-Dependent Group. (**a**) Fear in the waiting room, (**b**) fear upon entering the dental operatory, (**c**) fear during intravenous cannulation, and (**d**) fear when recalling the next treatment. Boxes represent the interquartile range (IQR); the horizontal line within each box indicates the median, and the whiskers show the minimum and maximum values.

For fear scores, VAS values were 40.0 (16.5–63.5) in the NSG and 35.0 (17.8–61.8) in the SDG while waiting in the reception area; 51.0 (18.0–83.0) and 42.0 (13.3–75.3) upon entering the dental operatory; 47.0 (7.5–82.0) and 42.5 (21.0–68.8) during cannulation; and 37.0 (7.5–54.0) and 33.5 (3.3–74.8) when recalling a hypothetical future dental visit after treatment completion, respectively. No statistically significant differences were observed between the groups at any time point (Figure 6).

#### 3.3.2. Measurement of Tension Scores

**Figure 7:**
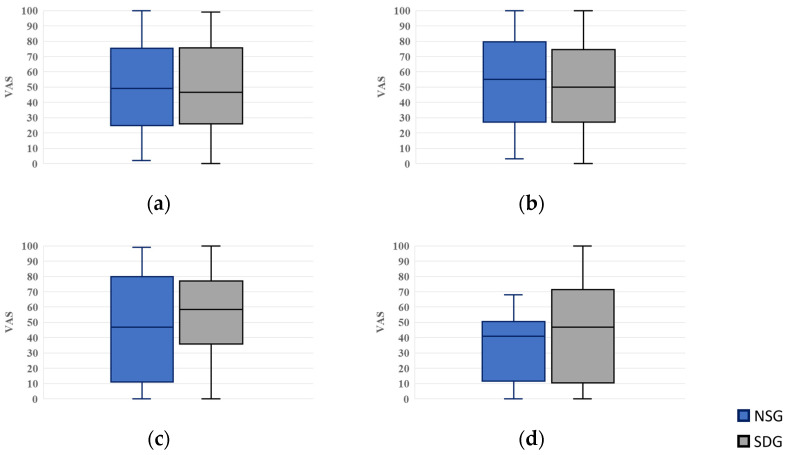
Box plots of visual analog scale (VAS) scores for tension. NSG: Non-Sedation Group; SDG: Sedation-Dependent Group. (**a**) Tension in the waiting room, (**b**) tension upon entering the dental operatory, (**c**) tension during intravenous cannulation, and (**d**) tension when recalling the next treatment. Boxes represent the interquartile range (IQR); the horizontal line within each box indicates the median, and the whiskers show the minimum and maximum values.

For tension scores, VAS values were 49.0 (25.0–75.5) in the NSG and 46.5 (26.0–75.8) in the SDG while waiting; 55.0 (27.0–79.5) and 50.0 (27.0–74.5) upon entering the operatory; 47.0 (11.0–80.0) and 58.5 (36.0–77.0) during cannulation; and 41.0 (11.5–50.5) and 47.0 (10.5–71.5) when recalling a hypothetical future dental visit after treatment completion, respectively. No statistically significant differences were observed between the groups at any time point (Figure 7).

#### 3.3.3. Measurement of Anxiety Scores

**Figure 8:**
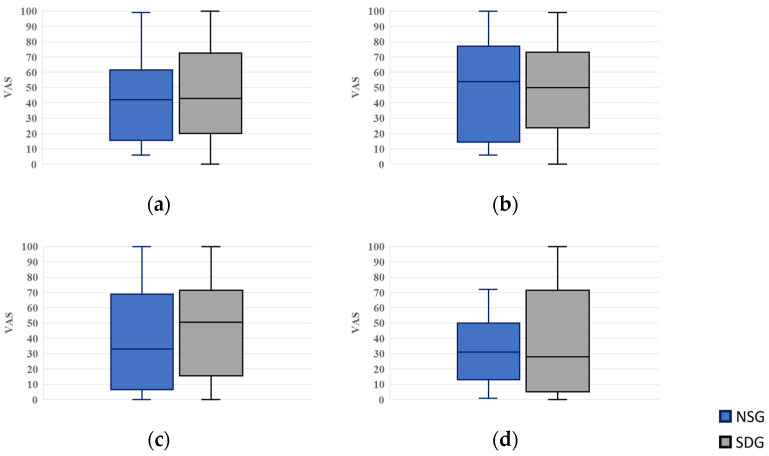
Box plots of visual analog scale (VAS) scores for anxiety. NSG: Non-Sedation Group; SDG: Sedation-Dependent Group. (**a**) Anxiety in the waiting room, (**b**) anxiety upon entering the dental operatory, (**c**) anxiety during intravenous cannulation, and (**d**) anxiety when recalling the next treatment. Boxes represent the interquartile range (IQR); the horizontal line within each box indicates the median, and the whiskers show the minimum and maximum values.

For anxiety scores, VAS values were 42.0 (15.5–61.5) in the NSG and 43.0 (20.0–72.5) in the SDG while waiting; 54.0 (14.5–77.0) and 50.0 (23.8–73.0) upon entering the operatory; 33.0 (6.5–69.0) and 50.5 (15.5–71.5) during cannulation; and 31.0 (13.0–50.0) and 28.0 (5.0–71.5) when recalling a hypothetical future dental visit after treatment completion, respectively. No statistically significant differences were observed between the groups at any time point (Figure 8).

#### 3.3.4. Measurement of the Evaluation of Attitudes, Satisfaction, and Expectations Toward Treatment

For the willingness to postpone future appointments, the VAS scores were 7.0 (0–49.0) in the NSG and 2.5 (0–13.3) in the SDG, with no statistically significant differences between the groups.

For treatment satisfaction, scores were 84.0 (61.5–91.0) in the NSG and 90.0 (84.5–96.8) in the SDG, with the SDG exhibiting significantly higher values (*p* = 0.015) and a small-to-moderate between-group effect size (*r* = 0.34, 95% CI: 0.08–0.59).

**Figure 9:**
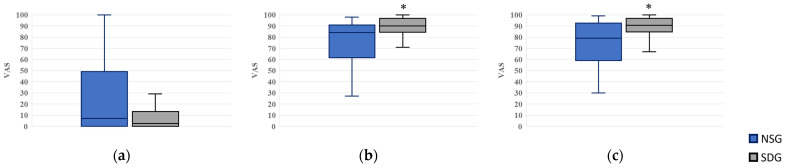
Box plots of visual analog scale (VAS) scores for the evaluation of attitudes, satisfaction, and expectations toward treatment. NSG: Non-Sedation Group; SDG: Sedation-Dependent Group. (**a**) Willingness to postpone the treatment appointment, (**b**) satisfaction with the current treatment, and (**c**) expectation for future treatment. Boxes represent the interquartile range (IQR); the horizontal line within each box indicates the median, and the whiskers show the minimum and maximum values. An asterisk (*) denotes a statistically significant difference between the groups (*p* < 0.05).

For expectations regarding future dental treatment, values were 79.0 (59.0–92.5) in the NSG and 90.5 (84.8–96.8) in the SDG. Scores were again significantly higher in the SDG (*p* = 0.022), with a small-to-moderate between-group effect size (*r* = 0.32, 95% CI: 0.07–0.56) (Figure 9).

Across all VAS measures of fear, tension, and anxiety, no statistically significant between-group differences were observed, despite the presence of between-group differences in physiological stress markers, indicating a dissociation between physiological autonomic measures and self-reported subjective experiences in this exploratory analysis.

## 4. Discussion

The classification into the NSG and SDG was performed post hoc based on the observed treatment course. Group membership therefore represents an outcome-defined clinical category—specifically, whether IVS could be discontinued—and should not be interpreted as a causal, explanatory, or mechanistic variable. Accordingly, this study does not seek to identify determinants or predictors of SDG status or to infer causal pathways, nor does it aim to use salivary α-amylase for prediction, but instead provides a strictly descriptive and exploratory comparison of physiological and subjective responses across treatment outcomes.

In this study, patients with dental phobia who required IVS were divided into the NSG and SDG for comparison. The results demonstrated that sAA activity was significantly higher in the SDG than in the NSG at both T0 and T1, and although a similar trend persisted at T2, the difference was not significant. These findings indicate higher sAA activity in the SDG than in the NSG at T0 and T1, representing observed differences in a selected physiological measure during anticipatory phases. sAA is a non-invasive physiological indicator that has been reported to be associated with activation of the SAM axis [14]. It has been increasingly used worldwide as a stress assessment tool in psychiatry, emergency medicine, and anesthesiology. In particular, in situations such as this study, where unconscious fear or anxiety is difficult to assess subjectively, sAA and HR were examined as physiological correlates that may accompany, but do not necessarily parallel, subjective emotional states during dental treatment. Continuation or discontinuation of IVS represents a behavioral–clinical outcome shaped by patient preference, perceived safety, and clinical judgment, rather than a direct physiological consequence of autonomic stress markers. The observed intergroup differences in sAA levels may reflect variability in autonomic arousal during anticipatory phases, rather than a uniform stress response. Notably, the lack of concordance between physiological measures (sAA, HR) and subjective ratings (VAS) indicates that the present findings should be interpreted as reflecting partial and dissociated response patterns rather than a single, integrated stress response. Accordingly, dependence on or withdrawal from IVS should be understood as a behavioral–clinical outcome influenced by patient preference, clinician judgment, and reinforcement processes, rather than as a direct physiological endpoint. In this exploratory study, sAA should not be regarded as a predictive biomarker or a central explanatory variable, but rather as one component among several physiological measures that may provide complementary, non-decisional information and warrants cautious interpretation and further investigation.

The continuation or discontinuation of IVS represents a complex behavioral–clinical trajectory influenced by multiple factors, including patient preference, perceived safety, clinician judgment, and learning processes. Accordingly, the sAA differences observed in this study are best understood as descriptively observed physiological correlates accompanying different treatment courses rather than determinants or drivers of clinical outcomes. Although T2 was defined as the time point at which anticipatory anxiety was expected to be maximal, no significant between-group differences in sAA activity were observed at this stage. One possible explanation is that anticipatory stress responses may be more prominent during earlier phases, such as waiting or environmental transition (T0 and T1), rather than during the execution of a specific procedural task. In addition, individual differences in prior experience with venous cannulation, coping strategies, and attentional focus during the procedure could contribute to increased response variability at T2, thereby attenuating detectable group-level differences. These findings suggest that stress reactivity in patients with dental phobia does not follow a uniform temporal pattern and underscore the exploratory and descriptive scope of the present analysis. Notably, treatment satisfaction and expectations for future treatment were higher in the SDG, which may be interpreted as reflecting the perceived effectiveness and procedural comfort of IVS within this group, rather than as evidence of a causal reinforcement mechanism. Taken together, although HR and VAS remain clinically relevant, sAA may offer complementary, non-decisional information regarding autonomic stress-related processes that are not fully captured by subjective or cardiovascular measures. It should also be noted that assessments related to “future treatment” reflect retrospective appraisals measured immediately after treatment completion, rather than prospective behavioral outcomes. Importantly, because group classification was performed post hoc based on observed treatment courses, all intergroup comparisons should be interpreted as descriptive, outcome-linked associations, rather than as causal or predictive relationships.

The intergroup differences in sAA activity may reflect differences in SAM axis reactivity. When confronted with fear-related stimuli, the sympathetic nervous system is known to be rapidly activated via the amygdala, inducing catecholamine release and subsequent sAA secretion from the salivary glands [27]. The higher sAA levels observed in the SDG may be interpreted as reflecting conditioned autonomic arousal to dental-related stimuli, among several possible mechanisms. Thus, elevated sAA should not be interpreted as a causal driver of sedation dependence but rather as a physiological correlate that may co-occur with the behavioral and cognitive processes underlying treatment preference and avoidance. In contrast, patients in the NSG may have undergone partial habituation or attenuation of conditioned autonomic responses through repeated treatment experiences, even if subjective anxiety was consciously perceived as manageable. Conversely, the lower sAA responses in the NSG may reflect, as one of multiple plausible interpretations, attenuation of fear-related autonomic responses through repeated treatment experiences, rather than definitive reductions in SAM axis reactivity. The HPA axis regulates prolonged stress responses through cortisol secretion, although its sensitivity to short-term changes is relatively low [27]. Although the present study did not directly evaluate HPA axis activity, the short-term intergroup differences detected in sAA should be interpreted cautiously and are insufficient to characterize overall stress physiology, but they may highlight individual variability in autonomic responses across different treatment courses.

HR is a general index of autonomic nervous system activity but is highly influenced by interindividual and environmental factors. In this study, HR was significantly higher in the NSG than in the SDG at T0, but this difference disappeared at T1 and T2. As all measurements were taken prior to IVS administration, pharmacological influences were excluded from the study. Rather, the initial HR elevation may be attributable to individual differences in anticipatory anxiety, arousal level, posture, respiration, or subtle body movements upon arrival at the clinic. Moreover, compared with sAA, HR may be less sensitive to subtle autonomic fluctuations under certain conditions, which could partly account for the inconsistent intergroup differences observed in this study. These findings are consistent with previous reports indicating that HR fluctuations do not always correspond to subjective fear and anxiety [28].

The development and persistence of dental phobia have been discussed within the framework of learning theory. Previous painful or panic-inducing experiences may contribute to the formation of a conditioned association between dental treatment and fear. Subsequently, the temporary relief achieved through avoidance behavior may reinforce such associations via negative reinforcement, leading to their consolidation [29]. In the SDG, where treatment was possible only under sedation, avoidance learning may have remained prominent, although this interpretation cannot be directly confirmed in the present study. In contrast, NSG patients who successfully completed treatment without sedation may have experienced a sense of accomplishment—“I overcame it by myself”—which may be consistent with processes such as partial fear attenuation or cognitive reappraisal, rather than definitive fear extinction. From the perspective of memory reconsolidation theory, the formation and maintenance of fear responses are often conceptualized as being linked to conditioned learning and memory processes, and their modification has been proposed to involve neurophysiological mechanisms such as fear memory reconsolidation [30].

Deep sedation has been suggested to potentially limit opportunities for the behavioral and emotional modification of fear, although direct evidence remains limited. In contrast, successful treatment experiences conducted under conscious sedation, during which patients remain responsive, have been proposed to help reshape patients’ perceptions of dental treatment and may contribute to a reduction in fear responses. However, such effects are often considered transient, whereas psychological approaches such as exposure therapy [31] and cognitive behavioral therapy [32] have demonstrated more durable efficacy. Although midazolam sedation can temporarily alleviate anxiety, fear has been reported to commonly re-emerge months later after the procedure. In contrast, exposure therapy has been shown to sustain improvement [33], suggesting that long-term reduction of dental fear is generally thought to require concurrent psychological intervention. Furthermore, combining benzodiazepines with exposure therapy has not consistently demonstrated enhanced long-term effectiveness. Accordingly, IVS may be more appropriately conceptualized not as a definitive therapeutic measure but as an initial intervention that could potentially facilitate the transition toward gradual, exposure-based treatment in clinical practice. Nevertheless, the present study did not assess sedation depth, intraoperative responsiveness, or postoperative memory recall; therefore, the extent to which differences in sedation level influence these processes cannot be determined from the present data.

Furthermore, this finding may be consistent with the observation that patients with higher satisfaction with sedation tended to experience greater difficulty discontinuing IVS, although no causal inference can be made. One possible physiological explanation that has been proposed in the literature is that deeper levels of sedation may suppress SAM and HPA axis reactivity, thereby reducing opportunities for direct engagement with fear-eliciting stimuli and adequate fear processing; however, such mechanisms were not examined in the present study and remain speculative [34]. In contrast, successful treatment provided under conscious sedation has been hypothesized to involve an appropriate degree of autonomic activation, which may allow patients to re-encode fear-eliciting stimuli as experiences perceived as more manageable and controllable. These observations do not establish a therapeutic model, but rather highlight the conceptual relevance of considering how pharmacological sedation and psychological processes might interact, generating hypotheses for future investigation regarding IVS withdrawal.

Taken together, the present study illustrates both the potential utility and inherent limitations of IVS in the management of dental phobia. IVS can function as a tool for interrupting avoidance behaviors driven by dental fear and enabling safe treatment; however, it does not necessarily eliminate fear. Accordingly, future research could explore integrative approaches combining IVS with psychological interventions, such as gradual exposure therapy, cognitive behavioral therapy, relaxation techniques [35], and adjunctive sensory interventions including color-based visual stimuli [24,36], to better understand possible pathways toward sedation withdrawal. Moreover, sAA activity was used in this study to index certain aspects of SAM-axis responsiveness and may serve as a physiological indicator alongside subjective and cardiovascular measures; however, its clinical utility or applicability cannot be determined from the present exploratory data. Accordingly, sAA should not be regarded as a biomarker for monitoring, prediction, or clinical decision-making, and its role remains confined to descriptive and hypothesis-generating contexts pending validation in future prospective studies.

This study has several limitations. First, group classification was based on post hoc clinical outcomes, and potential confounders, such as learning history or psychiatric background, were not fully controlled. Second, the assessment parameters were limited to sAA, HR, and VAS; neither cortisol (reflecting HPA axis activity) nor standardized psychological questionnaires with established psychometric validity were included in the study. Third, the limited sample size (*n* = 51 total) raises concerns regarding statistical power and the generalizability of the findings. Specifically, the observed significant differences, even after Bonferroni correction, should be interpreted cautiously, as they may be susceptible to bias or represent exploratory findings arising from the small cohort. Taken together, these limitations indicate that the present findings should be regarded as strictly hypothesis-generating rather than confirmatory, and future studies employing larger samples, longitudinal designs, and integrated multidimensional psychological and neuroendocrine measures are warranted.

Although this study identified the possibility of IVS withdrawal, it does not imply that all dental procedures can be performed without sedation. In practice, patients who achieve IVS withdrawal are typically able to tolerate less invasive procedures, such as oral hygiene maintenance or minor restorative treatments. Nevertheless, receiving these treatments without sedation may broaden therapeutic options and may facilitate the implementation of regular SPT. Maintaining oral hygiene through SPT has been associated with not only long-term dental prognosis but also systemic health and overall QOL. Therefore, rather than setting the complete elimination of fear as the only therapeutic goal, the ability to undergo minimally invasive procedures without sedation may be conceptualized as a realistic and clinically meaningful intermediate milestone in the management of dental phobia.

## 5. Conclusions

In this study, patients with dental phobia followed different clinical treatment courses, namely continuation or discontinuation of IVS. These patients exhibited heterogeneous patterns of selected physiological stress-related responses during anticipatory phases of dental treatment. These findings indicate interindividual variability in specific physiological measures, rather than a unified or integrated stress response, associated with different clinical treatment courses in patients with dental phobia, despite comparable levels of subjectively reported fear, anxiety, and tension. However, given the exploratory design and outcome-based group classification, no causal, predictive, or decision-making inferences regarding IVS discontinuation can be drawn from these findings. Accordingly, the present results should be interpreted strictly as descriptive and hypothesis-generating. Further studies integrating physiological, psychological, and behavioral frameworks are required to elucidate the clinical and behavioral processes associated with, rather than determine, sedation discontinuation in patients with dental phobia.

## Figures and Tables

**Table 1:** Demographic characteristics of study participants. Continuous variables are reported as median (IQR).

Patient Characteristics	Total (*n* = 51)	
Continuous Variables	Median (IQR)	
	NSG (*n* = 25)	SDG (*n* = 26)
Age (years)	42.0 (34.0–55.0)	38.0 (34.8–47.3)
Height (cm)	169.5 (158.8–173.9)	163.5 (155.5–172.0)
Weight (kg)	55.9 (50.4–61.6)	55.7 (53.1–64.4)
Body Mass Index (BMI)	19.7 (19.3–21.6)	21.6 (20.5–23.6)
Categorical Variables		
Sex [Male/Female]	9/16	11/15

**Table 2:** Items of the Customized Modified Dental Anxiety Scale (CMDAS). Each item is rated on a five-point Likert scale (1 = not anxious at all, 5 = extremely anxious), with total scores ranging from 5 to 25. Reprinted with permission from Ref. [24]. Copyright 2025, The Authors.

Items
(1) Do you feel anxious when making a dental appointment?
(2) Do you feel anxious while waiting in the reception area before treatment begins?
(3) Do you feel anxious when instruments are inserted into your mouth?
(4) Do you feel anxious about surgical dental procedures?
(5) Do you feel anxious about receiving an anesthetic injection in your mouth?

Each item is rated on a 5-point Likert scale (1 = not anxious, 5 = extremely anxious).

## Data Availability

The data presented in this study are available from the corresponding author upon reasonable request. The data are not publicly available due to ethical and privacy restrictions.
